# Can acute respiratory distress syndrome be treated?

**DOI:** 10.4155/fmc-2021-0014

**Published:** 2021-03-16

**Authors:** John W Christman, Manjula Karpurapu, Dehua Pei

**Affiliations:** ^1^Department of Medicine, Division of Pulmonary, Critical Care & Sleep Medicine, Ohio State University Wexner Medical Center, Davis Heart & Lung Research Institute, Columbus, OH 43210, USA; ^2^Department of Chemistry & Biochemistry, The Ohio State University, Columbus, OH 43210, USA

**Keywords:** ARDS, calcineurin inhibitors, COVID-19, peptidyl drugs

## Epidemiology & pathophysiology

The National Center for Advancing Translational Sciences considers the acute respiratory distress syndrome (ARDS) as a ‘rare disease’. The annual incidence of the ARDS is estimated at 64 to 79 cases per 100,000 population in the USA with a 27–43% mortality and a 56–64% long-term morbidity [1]. In 2020, approximately 33% of hospitalized COVID-19 patients developed ARDS and required transfer to an intensive care unit. The incidence of ARDS in nonsurvivors of COVID-19 is about 90% [2], indicating an overall incidence of fatal COVID-19 ARDS of nearly 400,000 in the first year of the pandemic. Appallingly, all together some 500,000 Americans have died of ARDS in the past year, which emphasizes the urgency of developing pharmacologic treatments based on the molecular mechanisms involved in ARDS.

Prior to the pandemic, the most prevalent cause of ARDS was bacterial sepsis, which is also the most common cause of mortality in hospitalized patients. In the winter, the incidence of ARDS rises as a result of the seasonal influenza outbreaks and subsequent post-influenza bacterial pneumonia. We are hopeful that COVID-19 ARDS can be reduced and ultimately eradicated by the recently released vaccines and universal vaccination, although this is not yet known and may be affected by viral mutation. Other relatively common causes of ARDS include aspiration of gastric contents, acute pancreatitis, massive blood transfusions and poly trauma. Although the overall mortality of ARDS has decreased over the decades due to multiple improvements in supportive care and respiratory management, effective treatments that are based on the molecular pathogenesis of ARDS have not yet been developed but are sorely needed.

Irrespective of the cause of ARDS, it is thought that a systemic cytokine storm initiates damage to the pulmonary microvascular endothelial and alveolar epithelial permeability barrier resulting in flooding of the alveolar space with protein-rich fluids. This process results in disruption of pulmonary gas exchange characterized by refractory hypoxemia requiring life supportive measures. This diffuse damage to the alveolar–capillary barrier is associated with further increased release of pro-inflammatory cytokines such as TNF-α, IL-1, and IL-6 [3]. These and other cytokines induce the expression of chemokines, such as IL-8 and CCL2, which subsequently recruit neutrophils and monocyte-derived macrophages to the lungs. Recruitment and activation of neutrophils is associated with release of injurious mediators such as reactive oxygen and nitrogen species and proteases. Extensive free radical production from this process overwhelms endogenous antioxidants and perpetuates oxidative cell damage in ARDS. Recruited monocyte-derived macrophages release additional inflammatory and counter inflammatory cytokines that contribute to the intensity and duration of ARDS inflammation, respectively. Recently, it has been discovered that activated neutrophils and macrophages also release injurious extracellular DNA/histones that contribute to lung inflammation [4]. This entire pathologic process is accentuated by secondary activation of platelets and the complement cascade, which further intensifies lung and systemic inflammation. Thus, induction of cytokine storm resulting in damage to the alveolar capillary barrier and dysregulation of innate immunity is believed to be the primary driver of the pathogenesis of ARDS.

## Management

Effective management of ARDS patients ideally involves a multidisciplinary team that includes a critical care physician, a critical care nurse, a respiratory therapist, a dietician, physical and occupational therapists, and a PharmD. Treatment for ARDS is focused on supporting oxygenation and ventilation without inflicting ventilator induced lung injury [5]. This is accomplished by ventilation with positive end expiratory pressure, which prevents lung injury induced by opening and closing of distal alveoli, referred to as atelectrauma. Ventilation using small tidal volumes (6 ml/kg ideal body weight) prevents volutrauma (i.e., overexpansion and damage of any remaining relatively normal alveoli). Positive pressure ventilation aims to keep the distending inspiratory pressure below 30 cm of water to prevent barotrauma. Finally, alternating patient positioning from supine to prone improve survival by resulting in a beneficial gravitational impact on lung infiltrates that minimizes regional physical forces and limits lung injury while improving oxygenation [6]. In extreme situations, ARDS patients are supported by extracorporeal membrane oxygenation [7].

Other effective management strategies include aggressive treatment of hypotension (shock) with fluids and vasopressors, avoidance of salt and water overload with diuretics and hemofiltration, and treatment of multiple organ failure with measures such as hemodialysis and transfusion of blood products. Additional helpful measures include the daily use of a checklist to ensure antibiotic stewardship, optimal use of sedation, prevention of deep venous thrombosis and pulmonary embolism, and nutritional support. Finally, physical and occupational therapy with early ambulation is beneficial for patients in the early recovery stages of ARDS.

## General pharmacologic therapies

There are no pharmacologic agents that specifically treat the underlying molecular pathophysiology of ARDS, although several drugs are widely used as supportive measures. For example, severely hypoxemic patients may be therapeutically paralyzed with neuromuscular blockers in combination with deep sedation to prevent ventilator dyssynchrony. Vasopressin and corticosteroids may be helpful adjunctive treatments for circulatory collapse, and broad-spectrum antibiotics are often necessary for treating any suspected or proven bacterial infections.

Since the first report of ARDS almost 50 years ago [8], many pharmacologic agents have been evaluated for treating ARDS. To date, a few have shown promise in animal models and early stages of clinical investigations, but none have been effective in phase III randomized clinical trials (RCTs); these include β2 adrenergic agonist therapy [9], early neuromuscular blockade [10], omega-3 fatty acids [11], rosuvastatin [12] and vitamin D [13]. The only agent that has shown clear clinical benefits is dexamethasone. In a randomized trial of 277 moderate to severe ARDS patients, treatment with dexamethasone showed an improvement in survival in the treatment group as compared with the placebo group over 60 days [14]. In another randomized trial of 299 COVID-19 patients with ARDS, dexamethasone increased the number of ventilator-free days over 28 days [15]. However, there is some concern that the prolonged use of high dosages of steroids can results in neuromuscular disability in some patients.

Vitamin C represents another promising but as yet inconclusive agent for treatment of ARDS. In a randomized trial of 167 patients with severe sepsis and acute respiratory failure, treatment with high doses of vitamin C failed to show significant improvement in the primary outcomes compared with the placebo group; however, a significant difference in the secondary outcomes was observed, including the 28-day mortality rate and the number of ventilator- and hospital-free days [16]. Similarly, treatment with allogeneic mesenchymal stromal cells in a small RCT of 60 patients with moderate to severe ARDS demonstrated safety but was inconclusive with respect to efficacy because of an unexpected cell viability issue [17].

## Early investigational therapies

### Low-dose carbon monoxide

In an early Phase 1 clinical trial of sepsis-induced ARDS, low-dose carbon monoxide was not associated with adverse events, but the study was not sufficiently powered for efficacy.

### Angiotensin-converting enzyme inhibitors

SARS-CoV-2 gains entry to lung cells through a mechanism that involves angiotensin-converting enzyme 2. Viral entry and multiplication are believed to disrupt the integrity of the alveolar capillary barrier leading to an increase in vascular permeability and pulmonary edema. An international multicenter RCT that examines the efficacy of angiotensin-converting enzyme inhibitors or angiotensin receptor blockers in COVID19 ARDS has been initiated, but results have not yet been reported [18].

### Anti-IL-6 antibodies

In an RCT of 243 hospitalized, moderately ill COVID19 patients with ARDS, treatment with tocilizumab showed no difference in preventing tracheal intubation or death, nor any difference in oxygenation at 5 or 14 days [19]. Although not yet peer reviewed, a recent report suggested that in a larger international multicenter RCT, tocilizumab or sarilumab resulted in a modest survival benefit.

### Calcineurin inhibitors

We and others have shown that depletion of macrophages by treatment with clodronate suppresses endotoxin-induced neutrophilic inflammation in mouse lungs. We further showed that upon treatment with lipopolysaccharides, activated NFATc3 regulates the expression of CCR2 and TNF-α in macrophages and Claudin-5 in pulmonary microvascular endothelial cells. These results suggested that pharmacologic inhibition of the NFATc3 function could provide an effective therapy for ARDS. We have recently developed a selective peptidyl inhibitor of the calcineurin-NFAT interaction, CNI103. CNI103 blocked NFATc3 activation in lung macrophages, decreased the production of TNF-α and IL-6, and prevented the development of sepsis-induced acute lung injury/ARDS in a mouse model [20]. *In vitro/ex vivo* studies demonstrate that CNI103 readily enters macrophages, monocytes and neutrophils but less efficiently T or B cells, suggesting that CNI103 may attenuate the inflammatory responses to viral infection without blocking T- and B-cell-mediated viral clearance. Our initial investigations indicate that CNI103 is well tolerated with limited, if any, toxicity. We are hopeful that CNI103 or a derivative will be tested in a future RCT.

## Summary

ARDS is a common and highly lethal condition. The mortality has improved with supportive care over the past few decades, but the morbidity has not. Although there is no current treatment for ARDS that is based on molecular mechanisms, the science of ARDS is improving rapidly. The current pandemic has emphasized the urgent need for treatments that reduce the mortality and alleviate the morbidity of ARDS.
